# Association of kidney function with posterior reversible encephalopathy syndrome in children 

**DOI:** 10.5414/CN110706

**Published:** 2022-02-10

**Authors:** Shruti M. Shah, Andrew M. South

**Affiliations:** 1 Wake Forest School of Medicine,; 2 Section of Nephrology, Department of Pediatrics, Brenner Children’s Hospital, Wake Forest School of Medicine,; 3 Department of Epidemiology and Prevention, Division of Public Health Sciences, Wake Forest School of Medicine, and; 4 Center for Biomedical Informatics, Wake Forest School of Medicine, Winston Salem, NC, USA

**Keywords:** acute kidney injury, blood urea nitrogen, hypertensive crisis, hypertensive encephalopathy

## Abstract

Aims: Investigate if kidney function markers predict posterior reversible encephalopathy syndrome (PRES) in children. Materials and methods: In a case-control study of high-risk children with confirmed PRES (n = 35) compared to controls (n = 14), we recorded blood urea nitrogen (BUN), serum creatinine, serum albumin, hemoglobin concentrations, estimated glomerular filtration rate, and documentation of acute kidney injury (AKI). We applied multivariable regression models and determined receiver operating characteristic curves. Results: Mean age was 9.5 (SD 4.9) years, 51% were female, 29% had chronic kidney disease, 67% had nephrotoxic medication exposure, and 29% had AKI. A 1-mg/dL increase in BUN (adjusted OR 1.03, 95% CI 0.99 – 1.07) and AKI (adjusted OR 3.78, 0.68 – 21.13) were minimally, but not statistically significantly, associated with PRES. BUN = 21.6 mg/dL performed best but had low ability to predict PRES (area under the curve 0.664, 0.498 – 0.831), with 60.0% sensitivity, 71.4% specificity, and positive and negative predictive values of 84.0% and 41.7%, respectively. Conclusion: Kidney function may be a relatively more minor risk factor for PRES than previously believed. Further prospective studies with larger sample sizes and better kidney function assessments are warranted to evaluate the role of kidney function in the development of PRES.

## Introduction 

Posterior reversible encephalopathy syndrome (PRES) – consisting clinically of seizures, headaches, altered mental status, and visual disturbances as well as neuroimaging findings of bilateral vasogenic edema in watershed areas affecting mainly the parietal and occipital lobes – likely results from disruption of normal cerebral vascular autoregulation and occurs in children as well as adults [1, 2]. Unlike most neurological disorders, PRES can reverse completely with removal or treatment of the underlying cause [2, 3]. However, PRES can cause permanent neurologic injury, such as intracranial hemorrhage, increased intracranial pressure, and death [4]. 

We previously observed that change in blood pressure (BP) over time strongly predicted PRES [5]. However, other risk factors for PRES, such as kidney disease are not well defined, especially in children [1, 5, 6, 7]. Albumin and hemoglobin contribute to tissue perfusion – especially in the setting of acute illness –, but little is known about their contributions to PRES. As the majority of children with hypertension (HTN) do not develop PRES, and some children who develop PRES do not have HTN, it is imperative to better define how kidney function affects PRES [2, 6]. 

Our objective was to investigate the association between several clinical markers of kidney function and PRES development. We hypothesized that kidney function, presence of acute kidney injury (AKI), serum albumin, and hemoglobin would be associated with and predict PRES in high-risk children. 

## Materials and methods 

### Study design and population 

This was a case-control study of hospitalized children at a single institution [5]. The Institutional Review Boards at Stanford School of Medicine and Wake Forest School of Medicine approved this study and deemed this retrospective data collection exempt from written informed consent and assent. We identified patients aged < 18 years admitted to a tertiary care hospital by searching a clinical data warehouse for documentation of PRES in brain magnetic resonance imaging (MRI) reports. We collected data from the electronic health record and clinical data warehouse and performed manual chart review to confirm data accuracy [8]. We excluded those patients who lacked sufficient clinical or MRI data to confirm PRES. We defined PRES by documentation of headache, seizures, altered vision, or mental status changes and established MRI criteria as previously described [5]. Our goal was to evaluate patients at high risk, so we defined as control participants those suspected of having PRES but whose MRIs were negative for PRES. 

### Data collection 

We recorded demographic and clinical information at hospital admission and at the time most proximal to concern for PRES as previously described [5]. We recorded presence of current or past medications, including antimicrobials, antihypertensives, calcineurin inhibitors, corticosteroids, monoclonal antibodies, and albumin. We documented recent surgeries, laboratory data only most proximal to PRES, and time to PRES development. We defined fluid overload as > 5% increase in weight from admission to when PRES was suspected [9]. 

### Exposures 

We recorded blood urea nitrogen (BUN), serum creatinine (Cr), serum albumin, and hemoglobin as well as clinical documentation of AKI at the time of concern for PRES. We calculated the estimated glomerular filtration rate (eGFR) and BUN-to-Cr ratio [10]. We classified eGFR per Kidney Disease: Improving Global Outcomes criteria [11, 12]. We defined high BUN > 20 mg/dL and high BUN-to-Cr > 30 mg/mg, based on established normative data [13, 14]. We defined hypoalbuminemia < 3.5 g/dL, the lower limit of the normal range, and severe hypoalbuminemia < 2 g/dL [15, 16]. Anemia was defined as hemoglobin concentration < 5^th^ percentile for age and sex: < 10.5 g/dL for 6 months – 2 years, < 11.5 g/dL for 2 – 12 years, < 13.0 g/dL for males 12 – 17 years, and < 12.0 g/dL for females 12 – 17 years [17]. We defined nephrotoxic medication exposure as having received carbapenems, vancomycin, aminoglycosides, penicillins, amphotericin, echinocandins, acyclovir, non-steroidal anti-inflammatory drugs, calcineurin inhibitors, cyclophosphamide, busulfan, cytarabine, pegaspargase, or fludarabine. 

### Statistical analysis 

We summarized our data with means with SD, medians with interquartile ranges, and frequencies with percentages. We used the χ^2^-test, Fisher’s exact test, t-test, and Wilcoxon rank-sum test to compare groups. We developed causal models of the relationships between kidney function and PRES a priori, inferred by the literature and our clinical expertise [18, 19]. We created individual directed acyclic graphs for each exposure-outcome model to evaluate potential sources of bias and included the minimally sufficient adjustment sets in our models to close off non-causal (i.e., biasing) pathways [19, 20]. These included:

- Chronic kidney disease (CKD) and nephrotoxic medication exposure for the Cr, BUN, BUN-to-Cr, and AKI models;
- CKD, eGFR, and albumin treatment for the serum albumin model;
- Age, sex, CKD, eGFR, fluid overload, and nephrotoxic medication exposure for the hemoglobin model.

Of note, each causal model best characterized BP as a mediator of the association of kidney function with PRES and did not include BP in the adjustment sets. We estimated the association of each exposure with PRES using individual bivariate and multivariable generalized linear models (logistic regression with logit link function and binomial distribution) and reported the OR and 95% CI. The multivariable models included the aforementioned adjustment sets. We employed bivariate and multivariable logistic regression models with Firth’s penalized likelihood estimates with the same adjustment sets when small-sample (i.e., rare-event) bias affected the maximum likelihood estimation [21]. 

We constructed individual receiver operating characteristic (ROC) curves for each exposure using multivariable logistic regression models. We calculated the areas under the curve, sensitivities, specificities, and positive and negative predictive values with corresponding 95% CI using the Wilson Score method due to the small sample size. We selected optimal cut points using the Youden Index to maximize the sum of sensitivity and specificity. A two-sided α of < 0.05 was statistically significant. SAS Enterprise Guide software for Windows was used (Version 7, SAS Institute Inc., Cary, NC, USA). 

## Results 

### Study population characteristics 

We identified 65 patients who had brain MRIs due to concern for PRES; 16 were excluded due to lack of adequate clinical or radiologic data. Of the included patients, 35 were cases, and 14 were controls. The mean age of the study population was 9.5 (SD 4.9) years, 51% were female, 47% identified as White, 29% Hispanic, 14% Asian, 8% Black, and 2% other (Table 1). 29% had CKD, and 67% had nephrotoxic medication exposure. Specific clinical and radiologic characteristics and outcomes of the cases were previously published [5]. Compared to controls, cases exhibited a significantly higher mean body mass index, systolic BP, and diastolic BP as well as a higher proportion of chronic HTN. 

### Kidney function 

**Figure 1:**
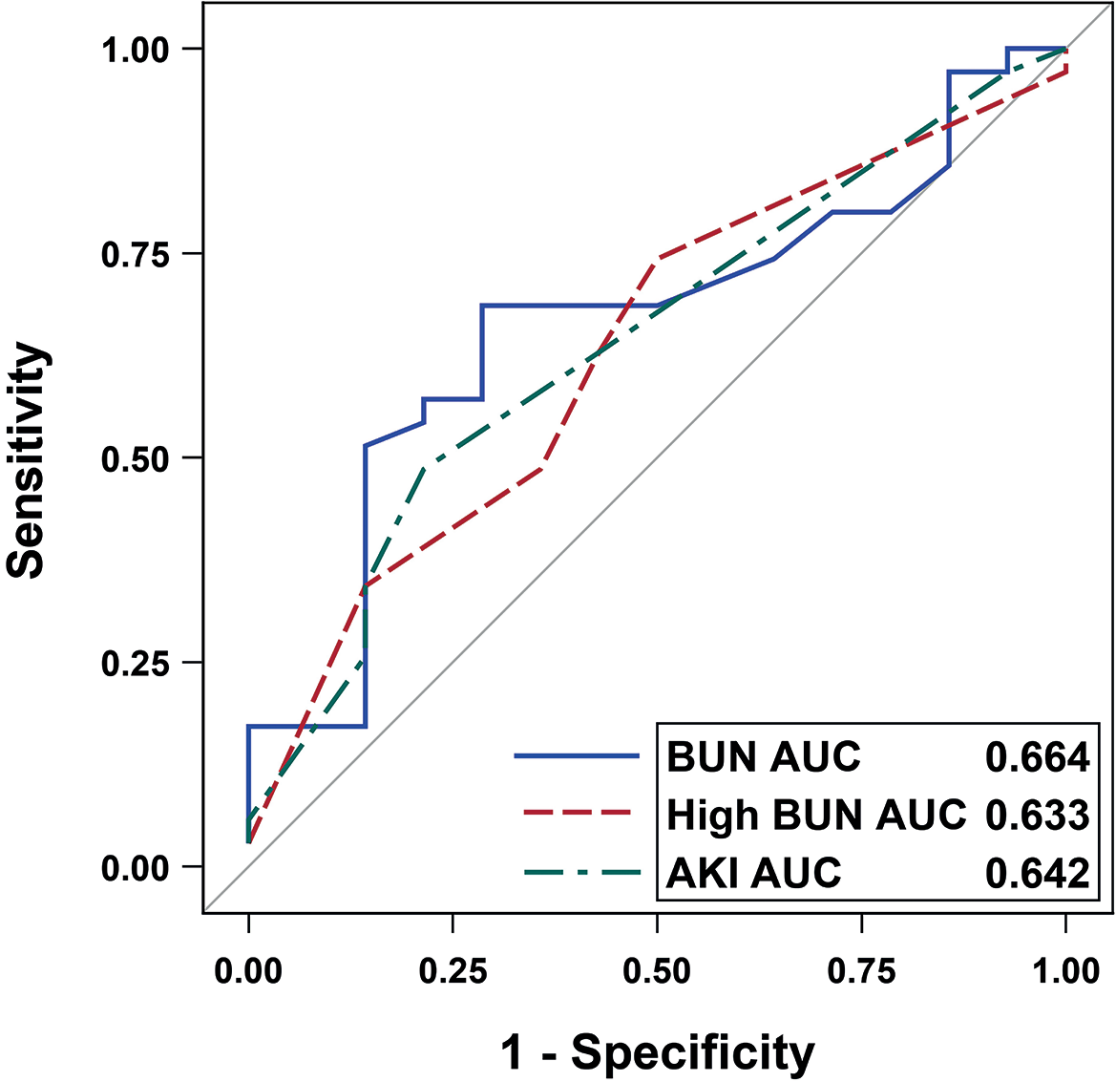
Receiver operating characteristic curves demonstrating that BUN = 21.6 mg/dL, BUN > 20 mg/dL, and AKI did not predict PRES. AUC = area under the curve; BUN = blood urea nitrogen; AKI = acute kidney injury; PRES = posterior reversible encephalopathy syndrome.

For the study population, 29% had AKI, median Cr was 0.7 mg/dL, and mean eGFR was 94.0 mL/min/1.73m^2^. On initial analysis, there were no statistically significant between-group differences in any kidney function measures (Table 2). Bivariate models demonstrated modest, though not statistically significant, associations for BUN, BUN > 20 mg/dL, AKI, and albumin (Table 3). Adjustment in the multivariable models confirmed that a 1-mg/dL increase in BUN (OR 1.03, 95% CI 0.99 – 1.07), BUN > 20 mg/dL (OR 2.95, 0.62 – 14.17), and AKI (3.05, 0.72 – 18.23, confirmed by Firth’s method) were not statistically significantly associated with PRES. Our predictive analyses showed that BUN, BUN > 20 mg/dL, and AKI weakly predicted PRES (Figure 1). An optimal BUN cut-off of 21.6 mg/dL performed best with 60.0% sensitivity and 71.4% specificity, with a positive predictive value of 84.0% and a negative predictive value of 41.7% (Table 4). 

## Discussion 

In a case-control study of children at high risk for PRES, we demonstrated that several markers of kidney function, including those related to BP regulation, were not statistically significantly associated with PRES. Our results suggest that kidney function may be a relatively minor risk factor for PRES. However, our results do not negate the importance of closely monitoring kidney function, including secondary parameters such as albumin, in high-risk patients. 

Among potential risk factors for PRES, AKI is well known to be involved in HTN pathophysiology. Renal vasoconstriction occurs commonly in high-risk patients due to a variety of factors such as sepsis and medication effects. This can acutely raise BP through several mechanisms, including increased antidiuretic hormone release and renin-angiotensin-aldosterone system activity, and subsequently can lead to cerebral vascular autoregulatory failure and vasogenic edema and ultimately PRES, especially with concurrent cerebral vascular endothelial injury [22, 23]. Further, patients with acute or chronic uremia are at risk for uremic encephalopathy, a toxic neurologic syndrome associated with delirium, myoclonus, chorea, and seizures that has been linked to PRES and which involves oxidative damage, hormone imbalances, neurotransmitter imbalances, and metabolic dysfunction [24, 25, 26]. However, hydration status, high-protein nutritional intake (enteral or parenteral), and underlying conditions, such as CKD, liver injury, or heart injury, can all confound BUN’s utility. 

In parallel with our prior findings and those of other investigators [5, 27], our results suggest that kidney function could have a differentially smaller effect on risk of PRES compared to that which HTN confers, though we caution against overly interpreting our results. One plausible reason for why these kidney function parameters were not associated with PRES is that BP could mediate this relationship [19]. Thus, future studies could incorporate this mediating role of BP in the study design and analysis. 

Strengths of our study include a relatively large sample size for this rare condition, mitigation of outcome misclassification, a control group with similar high-risk characteristics, and analysis of multiple kidney function markers that are relevant to BP control. We developed causal models and employed directed acyclic graphs, which allowed us to better characterize the relationships amongst our exposures, outcome, and potentially confounding factors to better identify and mitigate bias. Limitations of our study include patients from a single center only, case-control study design limiting our ability to fully address many sources of bias including confounding bias and selection bias, such as excluding patients who had PRES but who lacked sufficient clinical or radiological data to definitively diagnose or exclude PRES. We aired on the side of minimizing misclassification bias at the risk of having an increase in selection bias. Our small sample size may have not provided sufficient power to detect statistically significant between-group differences. There is also a lack of generalizability to patients with baseline CKD who are not admitted to the hospital or patients with AKI who lack additional PRES risk factors. We were not able to determine AKI definitively and instead relied on clinical documentation by the patients’ clinicians, rather than ICD-10 codes or KDIGO criteria. We were unable to characterize time-varying exposures, determine exact temporal relationships, or fully account for missing data to better characterize kidney dysfunction. We did not have information available on nephrotoxic medication dose or duration. 

## Conclusion 

In a case-control study of children at high risk for PRES, we found that kidney function parameters such as BUN and AKI were not statistically significantly associated with PRES. Future prospective cohort studies with larger sample sizes and more robust methods, including investigating BP as a mediator, are necessary to validate our findings and better delineate the role of kidney function in PRES development. 

## Acknowledgment 

We thank Abanti Chaudhuri, MD, and Scott Sutherland, MD, at Stanford School of Medicine for their assistance in conceptualizing this study and Julia Rushing and Tim Craven, M. Stat., at Wake Forest School of Medicine for their assistance with data analysis. Dr. Chaudhuri, Dr. Sutherland, Ms. Rushing, and Mr. Craven have no conflicts of interest. 

## Funding 

Spectrum Stanford Center for Clinical and Translational Research and Education (NIH/NCATS TL1TR001084), Wake Forest Clinical and Translational Science Award (NIH/NCATS UL1TR001420), and NIH/NHLBI K23HL148394 and L40HL148910. 

## Conflict of interest 

The authors have no relevant financial or non-financial interest to disclose. 

**Table 1.** Characteristics of cases compared to controls.

	Study population N = 49	Cases n = 35	Controls n = 14
Female	25 (51%)	19 (54%)	6 (43%)
Race/ethnicity			
White	23 (47%)	13 (37%)	10 (71%)
Hispanic	14 (29%)	12 (34%)	2 (14%)
Asian	7 (14%)	5 (14%)	2 (14%)
Black	4 (8%)	4 (11%)	0 (0%)
Other	1 (2%)	1 (3%)	0 (0%)
Age (years)	9.5 (4.9)	9.9 (4.4)	8.6 (6.1)
Height (cm)	129.8 (31.4)	131.5 (25.3)	125.4 (43.9)
Weight (kg)	36.4 (19.9)	38.0 (19.7)	32.4 (20.8)
Body mass index (kg/m^2^)*	19.8 (5.0)	20.6 (5.5)	18.0 (2.4)
Obesity	11 (22%)	10 (29%)	1 (7%)
Systolic BP (mmHg)*	147.1 (24.6)	156.0 (21.2)	124.9 (17.5)
Diastolic BP (mmHg)*	94.9 (22.4)	102.4 (20.9)	76.6 (14.2)
CKD	14 (29%)	10 (29%)	4 (29%)
Dialysis	8 (16%)	5 (14%)	3 (21%)
Diagnoses			
Solid organ transplant	19 (39%)	11 (31%)	8 (57%)
Other	11 (22%)	8 (23%)	3 (21%)
Stem cell transplant	5 (10%)	3 (9%)	2 (14%)
Systemic lupus erythematosus	5 (10%)	4 (11%)	1 (7%)
Blood malignancy	4 (8%)	4 (11%)	0 (0%)
Solid organ malignancy	3 (6%)	3 (9%)	0 (0%)
Glomerulonephritis	1 (2%)	1 (2%)	0 (0%)
Hemolytic uremic syndrome	1 (2%)	1 (3%)	0 (0%)
Known risk factors			
Steroids	36 (73%)	28 (80%)	8 (57%)
HTN*	35 (71%)	34 (97%)	9 (64%)
Calcineurin inhibitors	27 (55%)	17 (49%)	10 (71%)
Fluid overload	18 (37%)	14 (40%)	4 (29%)
Monoclonal antibodies	13 (27%)	10 (29%)	3 (21%)
Anti-HTN medication	35 (71%)	26 (74%)	9 (64%)
Calcium channel blocker	22 (45%)	15 (43%)	7 (50%)
Diuretic	21 (42%)	17 (48%)	4 (28%)
Alpha agonist	13 (27%)	10 (29%)	3 (21%)
ACE inhibitor	8 (16%)	7 (20%)	1 (7%)
Beta blocker	7 (14%)	6 (17%)	1 (7%)
Other	6 (12%)	5 (14%)	1 (7%)
ARB	2 (4%)	0 (0%)	2 (14%)
Albumin supplementation	10 (20%)	7 (20%)	3 (21%)
Blood transfusion	5 (10%)	4 (11%)	1 (7%)
Iron	7 (14%)	4 (11%)	3 (21%)
Epoetin use	5 (10%)	2 (6%)	3 (21%)

*p-value < 0.05 by Fisher’s exact test or t-test. Mean (SD) or n (%). ACE = angiotensin-converting enzyme; ARB = angiotensin II receptor blocker; CKD = chronic kidney disease; HTN = hypertension.

**Table 2.** Kidney function in cases compared to controls.

	Study population N = 49	Cases n = 35	Controls n = 14
eGFR (mL/min/1.73m^2^)	94.0 (54.6)	91.7 (54.4)	99.7 (56.5)
≥ 90 mL/min/1.73m^2^	28 (57%)	20 (57%)	8 (57%)
60 to < 90 mL/min/1.73m^2^	7 (14%)	5 (14%)	2 (14%)
30 to < 60 mL/min/1.73m^2^	5 (10%)	3 (9%)	2 (14%)
15 to < 30 mL/min/1.73m^2^	3 (6%)	2 (6%)	1 (7%)
< 15 mL/min/1.73m^2^	6 (12%)	5 (14%)	1 (7%)
Cr (mg/dL)	0.7 [0.5, 1.2]	0.9 [0.5, 1.2]	0.7 [0.5, 1.2]
BUN (mg/dL)	22 [14, 44]	25 [14, 50]	17 [14, 22]
BUN > 20 mg/dL	27 (55%)	21 (60%)	6 (43%)
BUN/Cr mg/mg	30.5 (19.1)	31.9 (21.5)	26.9 (10.9)
BUN/Cr > 30 mg/mg	22 (45%)	17 (49%)	5 (35%)
AKI	14 (29%)	12 (34%)	2 (14%)
Albumin (g/dL)	3 (0.8)	3.1 (0.7)	2.8 (1.0)
Hypoalbuminemia	29 (59%)	20 (57%)	9 (64%)
Severe hypoalbuminemia	5 (10%)	3 (9%)	2 (14%)
Hemoglobin (g/dL)	11.2 (2.2)	11.3 (2.0)	10.9 (2.8)
Anemia	27 (55%)	19 (54%)	8 (57%)

Mean (SD), median [interquartile range], or n (%). AKI = acute kidney injury; BUN = blood urea nitrogen; Cr = creatinine; eGFR = estimated glomerular filtration rate.

**Table 3.** Model results of the association of kidney function with PRES.

	Unadjusted		Adjusted	
	OR	95% CI	OR	95% CI
eGFR^a^	0.997	0.99 – 1.01	0.996	0.98 – 1.01
eGFR classification^a,b^	2.5	0.1 – 62.6	2.42	0.09 – 66.08
Cr^a^	1.28	0.8 – 2.05	1.38	0.8 – 2.4
BUN^a^	1.02	0.99 – 1.06	1.03	0.99 – 1.07
BUN/Cr^a^	1.02	0.98 – 1.06	1.03	0.98 – 1.08
BUN > 20 mg/dL^a^	2.0	0.57 – 7.02	2.95	0.62 – 14.17
BUN/Cr >30^a^	1.7	0.47 – 6.11	1.91	0.5 – 7.28
AKI^a^	3.13	0.6 – 16.33	3.78	0.68 – 21.13
Albumin^c^	1.65	0.76 – 3.61	1.7	0.73 – 3.93
Hypoalbuminemia^c^	0.68	0.1 – 4.77	0.66	0.07 – 6.43
Hemoglobin^d^	1.08	0.81 – 1.44	1.12	0.81 – 1.56
Anemia^d^	0.89	0.26 – 3.11	0.78	0.2 – 3.08

^a^Adjusted for CKD and nephrotoxic medication exposure; ^b^referent eGFR ≥ 90 mL/min/1.73m^2^; ^c^adjusted for CKD, eGFR, and albumin treatment; ^d^adjusted for age, sex, CKD, eGFR, fluid overload, and nephrotoxic medication exposure. AKI = acute kidney injury; BUN = blood urea nitrogen; Cr = creatinine; CKD = chronic kidney disease; eGFR = estimated glomerular filtration rate.

**Table 4.** BUN, high BUN, and AKI modestly predict PRES.

Predictor	AUC (95% CI)	Sensitivity (95% CI)	Specificity (95% CI)	PPV (95% CI)	NPV (95% CI)
BUN 21.6 mg/dL	0.664 (0.498 – 0.831)	60.0% (43.6 – 74.5%)	71.4% (45.4 – 88.3%)	84.0% (65.4 – 93.6%)	41.7% (24.5 – 61.2%)
BUN >20 mg/dL	0.633 (0.465 – 0.8)	60.0% (43.6 – 74.5%)	57.1% (32.6 – 78.6%)	77.8% (59.2 – 89.4%)	36.4% (19.7 – 57.1%)
AKI	0.642 (0.479 – 0.805)	34.3% (20.8 – 50.9%)	85.7% (60.1 – 96.0%)	85.7% (60.1 – 96.0%)	34.3% (20.8 – 50.9%)

Models adjusted for chronic kidney disease and nephrotoxic medication exposure. AKI = acute kidney injury; AUC = area under the curve; BUN = blood urea nitrogen; NPV = negative predictive value; PPV = positive predictive value.
